# Prevalence, Causes, and Factors Associated with Visual Impairment and Blindness among Registered Pensioners in Ghana

**DOI:** 10.1155/2019/1717464

**Published:** 2019-10-07

**Authors:** Benjamin D. Nuertey, Kwesi Nyan Amissah-Arthur, Joyce Addai, Victor Adongo, Augustine D. Nuertey, Clement Kabutey, Isaac Asimadu Mensah, Richard Bekoe Biritwum

**Affiliations:** ^1^Tamale Teaching Hospital, Tamale P. O. Box TL 16, Tamale, Ghana; ^2^Community Health Department, School of Public Health, University of Ghana, Korle-Bu, Ghana; ^3^Ophthalmology Unit, Department of Surgery, Korle Bu Teaching Hospital, College of Health Sciences, School of Medicine and Dentistry, University of Ghana, Accra, Ghana; ^4^Korle-Bu Teaching Hospital, Korle-Bu, Ghana; ^5^St Francis Xavier Hospital, Assin Fosu, Ghana; ^6^Nursing and Midwifery Training College, Koforidua, Ghana

## Abstract

**Purpose:**

To determine the prevalence, causes, and factors associated with presenting visual impairment and blindness among pensioners.

**Design:**

A nationwide cross-sectional study. This study was part of the analysis on data obtained in the pensioners' medical survey conducted among members of the National Pensioners Association in Ghana.

**Method:**

(i) Setting: it was a multicenter study involving thirteen centers throughout Ghana with a center in each regional capital. (ii) Study population: the study involved 4813 pensioners. (iii) Observation procedures: data were captured through the use of questionnaires, physical examinations including eye examinations, and urine and blood sample analysis. (iv) Main outcome measure: presenting visual impairment and blindness (as defined by the WHO ICD-10 classification).

**Results:**

The overall prevalence of blindness among pensioners in Ghana was 3.8% (95% CI = 3.2–4.4), while the prevalence of moderate and severe visual impairment was 21.7% (95% CI = 20.5–23.0). The prevalence of blindness was lowest in the 60–65-year-old age group (2.1% (95% CI = 1.3–2.8)) and highest in the above 80-year-old age group (12.2% (95% CI = 6.6–17.8)). Cataract was the leading cause of blindness (62.4%) and moderate and severe visual impairment (55.7%). Factors significantly associated with blindness and visual impairment include educational status, vegetarianism, arthritis, and having proteins in urine.

**Conclusion:**

There is a high prevalence of visual impairment and blindness among the pensioners in Ghana. Sadly, the greatest cause was cataract, which is correctable. Increase in formal education status will be important in the prevention of blindness and visual impairment.

## 1. Introduction

Visual impairment due to any cause is a major cause of significant morbidity, and it affects many globally [1]. In the year 2010, it was estimated that 285 million people were visually impaired worldwide, of which 39 million were blind and 246 million had low vision [2]. It was estimated that, without intervention, the prevalence of blindness might reach 76 million by the year 2020 [3]. In the elderly, aged 50 years and above, the global prevalence of blindness was estimated at 1.9% with moderate and severe vision impairment (MSVI, <6/18–3/60) estimated at 10.4% with about 31 million of global 36 million blind people within this age group [4, 5]. However, with the introduction of the global initiative to eliminate avoidable blindness (vision 2020: the right to sight), many agree that the initiative is in the right direction to reduce the prevalence of avoidable blindness [6, 7]. In the WHO Africa region, the prevalence of blindness in the year 2010 was estimated as 7300 per million of which 81.7% was among those aged 50 years and above [2, 8]. In Ghana, the World Health Organization (WHO) Study on global AGEing and adult health (SAGE) wave one which was conducted between 2007 and 2010, estimated the prevalence of low vision among adults 50 years or more to be 12.9% [9]. However, population-based studies in sections of the country among those aged 40 years and over had reported blindness prevalence ranging from 1.2% to 4.4%, with one study reporting a visual impairment prevalence of 17.1% [10, 11]. The current findings from the Ghana blindness and visual impairment study 2015 show that 19.12% of those aged 80 and above are blind [12].

There were varied underlying causes of visual impairment and blindness. It was estimated in the year 2010 that about 65% of blindness and 76% of the people with moderate/severe visual impairment worldwide were from preventable causes [13]. Recent studies reported cataract as the leading cause of blindness worldwide [14–16]. The leading cause of visual impairment, however, was uncorrected refractive error [1, 2]. Regardless of its cause, visual impairment or blindness was associated with reduced quality of life [17].

The aim of this study was to determine the current prevalence, causes, and factors associated with visual impairment and blindness among pensioners in Ghana. This study used the current WHO ICD 10^th^ revision recommendation of presenting visual acuity in the best eye to classify study participants. The study reported on the prevalence, causes, and factors associated with blindness and visual impairment.

## 2. Method

### 2.1. Study Design

A cross-sectional study was conducted with study participants from all ten regions of Ghana. Members of the National Pensioners Association (NPA) took part in the survey. It involved questionnaire interviews and physical examinations as well as laboratory examinations of urine and blood samples collected from study participants. The survey was conducted as part of a registration exercise for all members of the National Pensioners Association for the start of a Pensions Medical Scheme (PMS) [18]. The scheme was aimed as a top-up health insurance scheme for the members of the National Pensioners Association. It is suggested that the medical scheme would finance health care expenses of members of the National Pensioners Association in instances where the health care claim is outside the coverage of the National Health Insurance Scheme (NHIS). It was a nationwide exercise that took place from April to December 2014. Members of the National Pensioners Association converged at the registration/medical screening centers within the days of the screening.

### 2.2. Study Sites

The study took place in thirteen sites of which ten were in the regional capitals of the ten regions of Ghana, West Africa. The study settings were mainly urban. However, participants from nearby rural areas of each of the study sites also took part in the study. All the studies took place in thirteen sites across the country Ghana, with at least one site per the ten regions of Ghana.

### 2.3. Study Population and Eligibility Criteria

The study subjects were pensioners, and the majority of the pensioners were qualified as elderly as defined by the United Nations classification of 60 years and above [19]. There were some participants below 60 years who retired before the mandatory retirement age of 60 years due to disability and other reasons thereby, qualifying as pensioner. All pensioners who were members of the National Pensioners Association were eligible as participants of the study. Within the study period, 4813 members of the National Pensioners Association presented for the medical screening. The participant must be a member of the National Pensioners Association and must be a resident in Ghana.

### 2.4. Sample Size and Sampling Method

The aim was to screen all the members of the National Pensioners Association within and around the study sites. The estimated total number of eligible pensioners in and around the study sites was about 5000. Of these 4813 pensioners took part in the study.

### 2.5. Definitions and Eye Examinations

The Snellen chart in standard good illumination was used at a distance of six meters to measure the visual acuity. The tumbling “E” chart was used in instances that the pensioner had no formal education. Mild or no visual impairment was defined in this study as presenting distance visual acuity in the better eye equal to or better than 6/18 (20/70, 3/10, 0.3), which was consistent with the WHO ICD-10 category zero. Moderate visual impairment was defined as presenting distance visual acuity in the better eye worse than 6/18 but better or equal to 6/60 (20/200, 1/10, 0.1) corresponding to category 1 of the ICD-10 H54 categorization. Severe visual impairment was defined in the study as presenting distance visual acuity in the better eye worse than 6/60 but better or equal to 3/60 (20/400, 1/20, 0.05) corresponding to category 2 of the WHO ICD-10 H54 categorization. Moderate visual impairment and severe visual impairment were put together in the final analysis of this study and termed moderate and severe visual impairment (MSVI).

Blindness corresponding to category 3, 4, and 5 in the ICD-10 H54 categorization was put together in this study and termed blindness. Blindness was therefore defined as presenting distance visual acuity worse than 3/60. Additionally, examination of the anterior and posterior segments using a direct ophthalmoscope was performed. For other causes of visual impairment and blindness, we used the definitions from the 10th revision of International Statistical Classification of Diseases, injuries, and causes of death (ICD-10).

### 2.6. Study Materials and Data Capture Tool

Study questionnaires recorded sociodemographic data of participants. The past medical history of participants was collected. As was information on allergies, alcohol consumption, smoking, exercise, and diet. There was a physical examination form attached, which was used to capture data of the physical examination conducted by medical officers. Weight was measured with a weighing scale and height with a stadiometer. Blood pressure was measured using a standardized and validated electronic sphygmomanometer with appropriate cuff sizes. Visual acuity was checked using a Snellen chart and the tumbling “E” chart. Blood sample was taken, and a glucometer was used to measure the random blood sugar. Blood sample was collected into a serum separator bottle and transported to a laboratory for measurement of serum cholesterol using automated and standardized techniques.

### 2.7. Data Collection

Trained research assistants filled the questionnaire based on the response of the study participants. The weight was measured to the nearest one-kilogram, while the height to the nearest one-millimeter. Random blood sugar was measured using a glucometer. Medical officers perform physical examination, while the eye team checked the vision and carried out the comprehensive eye examinations. Personalized reports of the medical screening were sent to each participant.

### 2.8. Data Processing and Analysis

The data generated in the research were entered into EPIDATA 3.1 and exported into STATA/MP 11.0 (copyright 2004–2009) for analysis. The primary outcome in the study was presenting visual impairment and blindness. With regard to social class, the participants' previous occupation was classified under various social class headings according to the Registrar General's occupational classification of England and Wales [20]. The background characteristics of the respondents were obtained by cross tabulation. Causes of the visual impairment and blindness were tabulated based on the findings of the comprehensive eye exam. Causes with smaller frequencies and in instances with indeterminate causes were grouped together and termed “others.” Logistic regression was used to analyze the factors associated with blindness and visual impairment. First, the association between each of the potential factors and visual impairment/blindness was examined ignoring other variables. This analysis was important because it gave a fair idea as to which of the variables were strong predictors/related to visual impairment/blindness. Second, to construct a model with factors that were independently associated with visual impairment/blindness, and each of the independent variable was a candidate provided that the *P* value was 0.05 or less. A *P* value of 0.05 or less was considered statistically significant. With regards to the causes of moderate and severe visual impairment or blindness, multiple response analysis was carried out to rank the causes, and the results were tabulated as such.

### 2.9. Ethical Considerations

Review and approval was obtained from the National Pensioners Association board. The board further monitored each step of the data collection process. The content of the medical screening exercise was developed in extensive consultation with the executives of the National Pensioners Association. The study followed the tenets of the Declaration of Helsinki. Consent was voluntary, and each study participant had the right to withdraw at any stage of the study process. Uttermost privacy and confidentiality were maintained. No compensation or payments were made to any study participants. The results of the physical examination were carefully explained to all participants and were counselled on healthy lifestyle in old age. Personalized results of the study were sent to each participant in a sealed envelope to be given to his or her physician for further explanation. Data files were password protected. Hard copy data were stored in locked file cabinets, and access was limited to the principal investigator.

## 3. Results

### 3.1. Background Characteristics of the Study Participants

Table 1 displays the background characteristics of the study participants. Sixty-nine percent of the study participants were males, while 31% were females. The median age of the study participants was 66 (interquartile range = 7) years.

### 3.2. Prevalence of Presenting Blindness and Presenting Visual Impairment among Pensioners in Ghana

Table 2 displays the prevalence of moderate and severe visual impairment (MSVI) and the prevalence of blindness among study participants. The overall prevalence of blindness among pensioners in Ghana was 3.8% (95% CI = 3.2–4.4), while the prevalence of moderate to severe visual impairment was 21.7% (95% CI = 20.5–23.0). Thus, overall 256 per 1000 pensioners were either blind or visually impaired. The prevalence of blindness and visual impairment was worse in males. Thus, 22.6% (95% CI = 21.1–24.1) of the males were moderately and severely visually impaired (MSVI), whereas 19.7% (95% CI = 17.6–21.8) of the females were MSVI. Similarly, 4.5% (95% CI = 3.8–5.2) of the males were blind compared with 2.4% (95% CI = 1.5–3.2) among the females.

Generally, there was an increasing prevalence of blindness and moderate and severe visual impairment with increasing age. The prevalence of blindness was lowest in the below 65-year-old age group, thus 2.1% (95% CI = 1.3–2.8) and highest in the above 80-year-old group (12.2% (95% CI = 6.6–17.8)). Similarly, the prevalence of moderate and severe visual impairment (15.2% (95% CI = 12.4–17.0)) was lowest in the below 65-years age group and highest in the 80 years and above group (39.7% (95% CI = 31.3–48.1)). The prevalence of pensioners in Ghana who were either blind or MSVI was 173 per 1000 in the below 65-years age group, and it increased with increasing age to as high as 519 per 1000 in the 80 years and above group. The prevalence of blindness and MSVI generally decreases with increasing highest educational status. Pensioners who had no formal education had the highest prevalence of blindness and MSVI among the study participants.

The prevalence of MSVI and blindness among pensioners without any formal education was 31.7% (95% CI = 27.5–35.9) and 7.5% (95% CI = 5.1–9.9), respectively. The prevalence of MSVI and blindness among pensioners with tertiary education as highest educational status was 16.5% (95% CI = 14.3–18.7) and 2.8% (95% CI = 1.9–3.8), respectively.

Table 3 displays the age and sex specific prevalence of blindness and MSVI among the study participants. The prevalence of blindness in the males was higher than that among the female pensioners across the various age groups. The prevalence of blindness among male pensioners aged less than 65 years was 2.8 (95% CI = 1.7–3.9), whereas the prevalence among female pensioners for the same age group was 1.1 (95% CI = 0.3–1.8).

### 3.3. Causes of Presenting Moderate and Severe Visual Impairment (MSVI)

Table 4 displays the results of a multiple response analysis of the causes of moderate to severe visual impairment. The leading cause of MSVI was cataract. It accounted for about 55.7% of all the causes of MSVI. This was followed by refractive error accounting for 22.9%. Glaucoma was ranked the third highest cause of presenting MSVI among the pensioners accounting for 8.6% of all the causes of MSVI. Cataract surgery was fairly common. The other causes of MSVI were retinal diseases (4.1%), age-related macular degeneration (3.1%), cornea opacities (1.5%), optic nerve-related causes (1.1%), and other causes accounting for 3.0%.

### 3.4. Causes of Presenting Blindness among Pensioners in Ghana

Table 4 displays the causes of blindness among pensioners in Ghana. Uncorrected cataract accounted for more than half of all the causes of blindness among the pensioners. 62.4% of the presenting blindness was attributable to cataract. The second most common cause of blindness among pensioners in Ghana was glaucoma accounting for 12.9%. Retinal diseases followed with 4.8%. Age-related macular degeneration (3.2%), cornea opacities (2.7%), phthisis bulbi (2.7%), optic nerve related (2.2%), and others accounting for 9.1%.

### 3.5. Combined Causes of Presenting Visual Impairment and Blindness among Pensioners in Ghana

The leading cause of visual impairment classified as MSVI and blindness put together was cataract accounting for 52.3%. Refractive error (19.4%) and glaucoma (9.2%) also formed part of the top three causes of combined MSVI and blindness among the pensioners in Ghana. The other causes of the combined MSVI and blindness included retinal diseases (4.2%), cornea opacities (1.7%), optic nerve related (1.2%), phthisis bulbi (0.4%), and other causes accounting for 3.9%.

Factors associated with blindness and moderate and severe visual impairment among pensioners in Ghana.

In the unadjusted analysis, the odds of blindness and moderate and severe visual impairment among the males were 1.3 (95% CI = 1.1–1.5) times that among the females as shown in Table 5. Blindness and moderate and severe visual impairment (BMSVI) increased with increasing age such that the odds of BMSVI in pensioners aged 80 years and above were about 5.2 (95% CI = 3.5–7.5) times the odds of BMSVI among the pensioners below 65 years. Also, increasing highest educational status was associated with reduced risk of BMSVI. The odds of BMSVI among pensioners with no formal education were 2.7 (95% CI = 1.7–4.1) times the odds of BMSVI among the pensioners with the highest educational status as tertiary or vocational/technical training.

Body mass index was associated with BMSVI. Compared with the pensioners with normal BMI, being obese was associated with lower risk of presenting BMSVI (0.6, 95% CI = 0.5–0.9), whereas underweight was associated with an increased risk (1.7, 95% CI = 1.3–2.2) of being blind and moderately or severely visually impaired. Vegetarians were 1.8 (95% CI = 1.2–2.8) times likely to be blind and moderately or severely visually impaired compared to nonvegetarians. Social class was another factor associated with BMSVI. The social class V made up of unskilled according to the Registrar General social class classification was most associated with BMSVI (1.9, 95% CI = 1.3–3.6) compared to the professional group. Other factors like arthritis was marginally associated with BMSVI (1.2, 95% CI = 1.0–1.4). Raised serum lipids were associated with lower odds of BMSVI among the pensioners. Raised triglyceride (0.7, 95% CI = 0.5–1.0), low-density lipoprotein (0.8, 95% CI = 0.6–0.9), and total cholesterol (0.8, 95% CI = 0.6–0.9) were associated with reduced odds of BMSVI compared to having normal levels of triglyceride, low-density lipoprotein, and total cholesterol, respectively.

Having proteins in the urine was associated with BMSVI. Positive urine protein was associated with 1.5 (95% CI = 1.2–1.9) times the odds of BMSVI compared with the odds of having normal urine protein. Table 5 displays the findings from the logistic regression.

### 3.6. Adjusted Analysis

The adjusted odds ratios are displayed in Table 6. Model A adjusted for age and sex, while model B adjusted for age, sex, and highest educational status. Formal education was protective against BMSVI in the adjusted analysis compared with no formal education. Primary education was associated with 30% reduced risk of BMSVI (0.7, 95% CI = 0.5–0.9). Similarly, secondary education as the highest formal educational status was also associated with a reduced risk of BMSVI (0.7, 95% CI = 0.5–0.9).

Tertiary education and vocational/technical education as highest formal education was associated with reduced risk of BMSVI (0.6, 95% CI = 0.4–0.7) and (0.5, 95% CI = 0.3–0.8), respectively. Also, in the adjusted analysis, the odds of BMSVI were high in the underweight WHO BMI classification group (1.5, 95% CI = 1.1–1.9), whereas it was reduced in the WHO overweight group (0.8, 95% CI = 1.0–0.9). Vegetarians were 1.9 (95% CI = 1.2–3.0) times more associated with BMSVI compared to nonvegetarians. Pensioners having a history of arthritis were associated with BMSVI in the adjusted analysis (AOR = 1.2, 95% CI = 0.5–1.5) compared to pensioners with no such history. Urine proteins were associated with BMSVI. Pensioners with trace urine proteins were 1.3 (95% CI = 1.1–1.9) times more likely to be blind and moderately or severely visually impaired compared with those with normal urine proteins. Also pensioners with urine proteins greater than 30 mg/dl or 0.3 mmol/L were 1.5 (95% CI = 0.5–1.0) times more likely to have BMSVI.

## 4. Discussion

Age has been a significant factor that influenced the development of visual impairment and blindness. Age has been described as an important related factor in eye disease [21]. More than half (519 per 1000) of all pensioners above the age of 80 in Ghana have a visual impairment with 122 per 1000 pensioners 80 years and above blind. The above statistics is worrisome since majority of causes of blindness and visual impairment in Ghana were from preventable causes. According to findings from the Ghana blindness and visual impairment study 2015, more than half of blindness in Ghana was caused by cataract [12].

The prevalence of blindness and MSVI generally decreased with increasing highest educational status. Pensioners who had no formal education had the highest prevalence of blindness and MSVI among the study participants. Education and occupation have been used as proxy for socioeconomic status [22] which has been linked with visual impairment [23, 24]. It has been shown that people from high social classes have a stronger health care-seeking behavior [25]. The economic empowerment of pensioners with higher educational status means that they may be more likely to seek and undertake early treatment for eye conditions. In such circumstances, they were less likely to have presented at the study with MSVI and blindness due to prior intervention.

More than half of all blindness and visual impairment in this study can be directly linked to cataract. Cataract is the leading cause of blindness worldwide, and it is a multifactorial disease associated with several factors [26]. Cataract still remains a public health problem because of lack of skilled personnel and facilities to adequately handle cases of cataract that keeps rising due to population growth [21]. It has been estimated that close to 90% of all cases of cataract globally is from developing countries; however, most of the research into cataract are in the United States and the European countries [21].

Cataract is ranked as the leading cause of blindness and visual impairment [14], a situation that was also observed in Ghana. Glaucoma was ranked the second leading cause of blindness among the pensioners in Ghana and the third leading cause of MSVI among the pensioners. Increasing age was strongly associated with the risk of being blind and moderately or severely visually impaired. Age above 80 years was associated with about five times the odds of being BMSVI compared to that of the pensioners below 65 years. Several years of study on aging and vision have demonstrated the effects of aging and vision, which is true for almost all people from all nations including Ghana [27]. Globally, causes of blindness included age-related macular degeneration, glaucoma, corneal opacities, uncorrected refractive errors, and diabetic retinopathy [13]. In Ghana, the leading causes of blindness are from correctable causes such as cataract [12]. Causes of visual impairment included cataract, diabetic retinopathy, and age-related macular degeneration.

Nutritional factors were significantly associated with BMS visual impairment. Firstly, body mass index was found to be related with blindness and moderate and severe visual impairment. Those within the WHO BMI underweight category were about 50% more likely to be blind and moderately and severely (BMS) visually impaired. Also, analyzing the crude odds ratio, raised total cholesterol, raised low-density lipoprotein, and raised triglyceride levels were negatively associated with being BMS visually impaired. Finally, vegetarians were about twice likely to be BMS visually impaired compared to nonvegetarians. We can attribute nutritional causes to play a significant role in the development of visual impairment in the elderly.

Pensioners who were diagnosed as having any form of arthritis were more likely to be BMS visually impaired, and this may be due to the use of steroids for treating rheumatoid arthritis [28].

Also, obesity was found to confer less risk against the development of BMS visual impairment. We also noted that having proteins in the urine was associated with presenting BMS visual impairment in the elderly. This requires further studies to ascertain the nature of the proteinuria if it is of infective or renal pathology.

This study was done in pensioners; given the demographic of workers in Ghana, particularly those who have retired, there are more males in employment and subsequently more males in the pensioner category. This could also explain the higher prevalence of visual impairment in men than women, contrary to previous meta-analysis of various worldwide studies that revealed higher proportion of worldwide blindness in women than in men.

Limitations encountered in this study were that not all pensioners were members of the National Pensioners Association. Also, a significant proportion of pensioners whose previous work was in the informal sector were not members of the National Pensioners Association and such, the prevalence of blindness and visual impairment may be underestimated by this study. The setting of the study was mainly urban. Also, majority of the elderly people who had no formal education were not members of the National Pensioners Association [29] and as such, the prevalence of blindness obtained as 3.8% and the prevalence of visual impairment, which was estimated as 21.7%, may be lower than what really persist among the elderly population in Ghana. This is because pensioners without any formal education were more likely to be MSVI or blind compared with any category of elderly who had some formal education [30].

The authors recommend that the Eye Care Secretariat of the Ghana Health Service should include a strategy for identifying pensioners with visual impairment and prioritize their care. There will have to be a deliberate plan to seek out these individuals using the social security system to identify them. Given the relatively young population of the country, there will also have to be a strategy to forecast the increase in pensioner numbers over the next few decades as well as make provision for their eye care needs. Also, the role of the National Health Insurance Scheme (NHIS) and access to preventive eye care services in Ghana need a critical review because no one should theoretically be cataract blind.

This study shows that even though there is provision for cataract surgery under the National Health Insurance Scheme of Ghana, there is a high prevalence of visual impairment and blindness due to cataract, which appears to be worse in men of lower educational status.

## Figures and Tables

**Table 1:** Background characteristics of study participants.

Proportion by sex			
Participant characteristics	Female *n* (%)	Male *n* (%)	All participants *N* (column %)
All participants	1,482 (31.0)	3,300 (69.0)	4,782 (100)
Age in years			
60–65	704 (50.5)	960 (31.4)	1,664 (37.3)
66–69	461 (33.1)	1,056 (34.5)	1,517 (34.0)
70–74	166 (11.9)	627 (20.5)	793 (17.8)
75–79	50 (3.6)	295 (9.7)	345 (7.7)
≥80	14 (1.0)	123 (4.0)	137 (3.1)
Current marital status			
Never married	36 (2.6)	40 (1.3)	76 (1.7)
Married	556 (40.3)	2,679 (88.0)	3,235 (73.1)
Widow/widower	511 (37.1)	200 (6.6)	711 (16.1)
Divorced	157 (11.4)	63 (2.1)	220 (5.0)
Separated	119 (8.6)	62 (2.0)	181 (4.1)
Body mass index (by WHO BMI cutoff/classification)			
Underweight	34 (2.4)	232 (7.6)	266 (6.0)
Normal	393 (28.0)	1,743 (56.8)	2,136 (47.8)
Overweight	492 (35.1)	848 (27.6)	1,340 (30.0)
Obese	483 (34.5)	246 (8.0)	729 (16.3)
Highest formal educational status			
None	70 (5.4)	423 (15.1)	493 (12.0)
Primary	517 (39.7)	1,116 (39.9)	1,633 (39.4)
Secondary	195 (15.0)	434 (15.5)	629 (15.4)
Tertiary	452 (34.7)	724 (25.9)	1,176 (28.7)
Vocational	68 (5.2)	100 (3.6)	168 (4.1)
Known diabetic status			
Not a diabetic	1,167 (86.0)	2,745 (91.4)	3,910 (89.7)
Known diabetic	190 (14.0)	258 (8.6)	448 (10.3)
Known hypertension status			
Not a hypertensive	531 (38.3)	1,785 (58.6)	2,316 (52.2)
Known hypertensive	857 (61.7)	1,262 (41.4)	2,119 (47.8)

**Table 2:** Prevalence of presenting blindness and visual impairment among pensioners in Ghana.

	Mild or no visual impairment		Moderate to severe visual impairment		Blindness	
	*n* (%)	95% CI	*n* (%)	95% CI	*n* (%)	95% CI
Overall	3,300 (74.4)	73.1–75.7	964 (21.7)	20.5–23.0	170 (3.8)	3.2–4.4
Sex						
Male	2,236 (72.9)	71.3–74.5	693 (22.6)	21.1–24.1	138 (4.5)	3.8–5.2
Female	1,062 (80.0)	75.8–80.2	268 (19.7)	17.6–21.8	32 (2.4)	1.5–3.2
Age (years)						
<65	1,323 (82.7)	80.1–84.6	243 (15.2)	12.4–17.0	33 (2.1)	1.3–2.8
65–69	1,153 (78.2)	76.1–80.3	283 (19.2)	17.2–21.2	39 (2.64)	1.8–3.4
70–74	507 (66.1)	62.7–69.5	218 (28.4)	25.2–31.6	42 (2.5)	3.9–7.1
75–79	182 (54.8)	49.5–60.2	118 (35.5)	30.4–40.7	32 (9.6)	6.5–12.8
≥80	63 (48.1)	39.5–56.7	52 (39.7)	31.3–48.1	16 (12.2)	6.6–17.8
Highest formal educational status						
None	291 (60.8)	56.3–65.1	152 (31.7)	27.5–35.9	36 (7.5)	5.1–9.9
Primary	1,180 (74.8)	72.7–80.0	342 (21.7)	19.7–23.7	55 (3.5)	2.6–4.4
Secondary	466 (76.5)	73.1–79.9	123 (20.2)	17.0–23.4	20 (3.3)	1.9–4.7
Tertiary	915 (80.7)	78.4–83.0	187 (16.5)	14.3–18.7	32 (2.8)	1.9–3.8
Vocational	129 (80.6)	74.5–86.8	29 (18.1)	12.1–24.1	2 (1.3)	0.5–3.0
Body mass index WHO classification						
Underweight	152 (60.6)	54.5–44.4	86 (34.3)	28.4–40.2	13 (5.2)	2.4–7.9
Normal	1,493 (71.7)	69.7–73.6	491 (23.6)	21.8–25.4	99 (4.7)	3.8–5.7
Overweight	1,023 (78.7)	76.5–80.9	243 (18.7)	16.6–20.8	34 (2.6)	1.7–3.5
Obese	558 (79.8)	76.9–82.8	122 (17.5)	14.6–20.3	19 (2.7)	1.5–3.9
Current smoking status						
Nonsmoker	3,008 (80.0)	73.6–76.3	853 (21.3)	20.0–22.5	151 (3.8)	3.2–4.4
Smoker	66 (69.6)	60.2–78.8	23 (24.2)	15.5–32.9	6 (6.3)	1.4–11.2
Known diabetic status						
Nondiabetic	2,826 (74.8)	73.4–76.2	808 (21.4)	20.1–22.7	144 (3.8)	3.2–4.4
Diabetic	330 (76.4)	72.3–80.4	83 (19.2)	15.5–22.9	19 (4.4)	2.5–6.3
Region of residence						
Central	97 (58.1)	50.6–65.6	55 (32.9)	25.8–40.0	15 (9.0)	4.6–13.3
Upper West	218 (66.9)	61.8–72.0	82 (25.2)	20.4–29.9	26 (8.0)	5.0–10.9
Ashanti	937 (85.1)	83.0–87.2	123 (11.2)	9.3–13.0	41 (3.7)	2.6–4.8
Northern	218 (66.7)	61.5–71.8	87 (26.6)	21.8–31.4	22 (6.7)	4.0–9.4
Upper East	156 (59.1)	53.1–65.0	96 (36.4)	30.5–42.2	12 (4.6)	2.0–7.1
Brong-Ahafo	210 (75.8)	70.8–80.9	55 (19.9)	15.1–24.6	12 (4.3)	1.9–6.7
Western	415 (64.6)	60.9–68.3	208 (32.4)	28.7–36.0	19 (3.0)	1.6–4.3
Volta	346 (71.8)	67.8–75.8	126 (26.1)	22.2–30.1	10 (2.1)	0.8–3.3
Eastern	345 (87.6)	84.2–90.8	41 (10.4)	7.4–13.4	8 (2.0)	0.6–3.4
Greater Accra	313 (82.6)	78.8–86.4	62 (16.4)	12.6–20.0	4 (1.1)	0.03–2.0

**Table 3:** Age and sex specific prevalence of blindness and visual impairment among pensioners in Ghana.

Sex stratified by age	Mild or no visual impairment		Moderate to severe visual impairment		Blindness	
	*n* (%)	95% CI	*n* (%)	95% CI	*n* (%)	95% CI
Males stratified by age in years						
<65	758 (81.5)	79.0–84.0	146 (15.7)	13.4–18.0	26 (2.8)	1.7–3.9
65–69	807 (78.6)	76.0–81.1	191 (18.6)	16.2–21.0	29 (2.8)	1.8–3.8
70–74	407 (66.3)	62.5–70.0	174 (28.3)	24.8–31.9	33 (5.4)	3.6–7.2
75–79	156 (54.7)	48.9–60.5	99 (34.7)	29.2–40.3	30 (10.5)	7.0–14.1
≥80	59 (50.0)	40.9–59.1	46 (40.0)	30.1–47.8	13 (11.0)	5.3–16.7
Females stratified by age in years						
<65	565 (84.5)	81.7–87.2	97 (14.5)	11.8–17.2	7 (1.1)	0.3–1.8
65–69	345 (77.4)	73.4–81.2	91 (20.4)	16.7–24.2	10 (2.2)	0.9–3.6
70–74	100 (65.4)	57.8–72.9	44 (28.8)	21.6–36.0	9 (5.9)	2.1–9.6
75–79	26 (55.3)	40.9–69.7	19 (40.4)	26.2–54.6	2 (4.3)	1.6–10.1
≥80	4 (30.7)	4.6–56.9	6 (46.2)	17.9–74.4	3 (23.1)	0.7–46.9

**Table 4:** Causes of presenting moderate and severe visual impairment (MSVI) and presenting blindness among pensioners in Ghana.

Rank	Visual impairment among pensioners		Presenting blindness	
	Causes	*N* (%)	Causes	*N* (%)
1	Cataract	579 (55.7)	Cataract	116 (62.4)
2	Refractive errors	238 (22.9)	Glaucoma	24 (12.9)
3	Glaucoma	89 (8.6)	Retinal disease	9 (4.8)
4	Retinal disease	43 (4.1)	Age-related macular degeneration	6 (3.2)
5	Age-related macular degeneration	32 (3.1)	Cornea opacities	5 (2.7)
6	Cornea opacities	16 (1.5)	Phthisis bulbi	5 (2.7)
7	Optic nerve related	11 (1.1)	Optic nerve related	4 (2.2)
8	Others	31 (3.0)	Others	17 (9.1)

**Table 5:** Factors associated with blindness and moderate and severe visual impairment among pensioners in Ghana.

	Crude odds ratio	95% confidence interval	*P* value
Sex			
Male	1.3	1.1–1.5	<0.0005
Female	—	—	
Age in years			
<65	—	—	
65–69	1.3	1.1–1.6	<0.0001
70–74	2.5	2.0–3.0	
75–79	4.0	3.1–5.1	
≥80	5.2	3.5–7.5	
Highest formal education			
No formal education	2.7	1.7–4.1	<0.0001
Primary	1.4	0.9–2.1	
Secondary	1.3	0.8–2.0	
Tertiary	1.0	0.7–1.5	
Vocational	-	—	
Body mass index WHO classification			
Underweight	1.7	1.3–2.2	<0.0001
Normal	—	—	
Overweight	0.7	0.6–0.8	
Obese	0.6	0.5–0.9	
Vegetarian			
Nonvegetarian			
Vegetarian	1.8	1.2–2.8	<0.001
History of arthritis			
No arthritis			
Had arthritis	1.2	1.0–1.4	<0.05
Social class by previous occupation			
I (professional)	—	—	<0.0001
II (managerial/technical)	1.1	0.8–1.5	
III (N; skilled nonmanual)	1.0	0.7–1.3	
III (M; skilled manual)	1.3	0.9–1.8	
IV (partly skilled)	1.6	1.4–2.1	
V (unskilled)	1.9	1.3–2.6	
Serum triglyceride level			
Normal			
Raised	0.7	0.5–1.0	<0.05
Serum Ldl level			
Normal			
Raised	0.8	0.6–09	<0.05
Serum total cholesterol level			
Normal			
Raised	0.8	0.6–0.9	<0.05
Urine proteins			
Negative	—	—	<0.0005
Trace	1.3	1.1–1.7	
Positive (+, ++, +++, ++++)	1.5	1.2–1.9	

**Table 6:** Factors associated with blindness and moderate and severe visual impairment among pensioners in Ghana adjusting for age and sex in model (A) and adjusting for age, sex, and highest educational status in model B.

	^*∗*^Model A			#Model B		
	AOR ∧	95% CI^+^	*P* value	AOR ⋀	95% CI^+^	*P* value
Highest formal education						
No formal education	—					
Primary	0.7	0.5–0.9	<0.001			
Secondary	0.7	0.5–0.9	<0.005			
Tertiary	0.6	0.4–0.7	<0.001			
Vocational	0.5	0.3–0.8	<0.005			
Body mass index WHO classification						
Underweight	1.5	1.1–1.9	<0.05	1.5	1.1–2.1	0.006
Normal	—			—		
Overweight	0.8	0.6–0.9	<0.005	0.8	0.7–1.0	0.06
Obese	0.8	0.6–1.0	<0.05	0.8	0.6–1.0	0.9
Vegetarian						
Nonvegetarian	—			—		
Vegetarian	1.9	1.2–3.0	<0.005	1.9	1.2–3.0	0.009
History of arthritis						
No arthritis	—	—				
Had arthritis	1.2	1.0–1.5	<0.05	1.3	1.1–1.6	0.008
Urine proteins						
Negative	—	—		—		
Trace	1.3	1.1–1.7	<0.5	1.5	1.2–1.9	0.002
Positive (+, ++, +++, ++++)	1.5	1.2–1.9	<0.005	1.5	1.1–1.9	0.003

^*∗*^Model A: adjusting for age and sex. #Model B: adjusting for age, sex, and highest educational status. AOR^: adjusted odds ratio. 95% CI^+^: 95% confidence interval.

## Data Availability

The data used to support the findings of this study are available from the corresponding author upon request.
